# Supplementary material for: Causal Relationship between Aging and Anorexia Nervosa: A White-Matter-Microstructure-Mediated Mendelian Randomization Analysis
Source: Biomedicines. 2024 Aug 16;12(8):1874. doi: 10.3390/biomedicines12081874 (PMC11351342; doi:10.3390/biomedicines12081874)
Supplement: Supplementary file 1 [file biomedicines-12-01874-s001.zip › Supplementary Figure/Figure S9/Leaveoneout plot of MR analysis for ubm-b-1917 to AN.pdf]

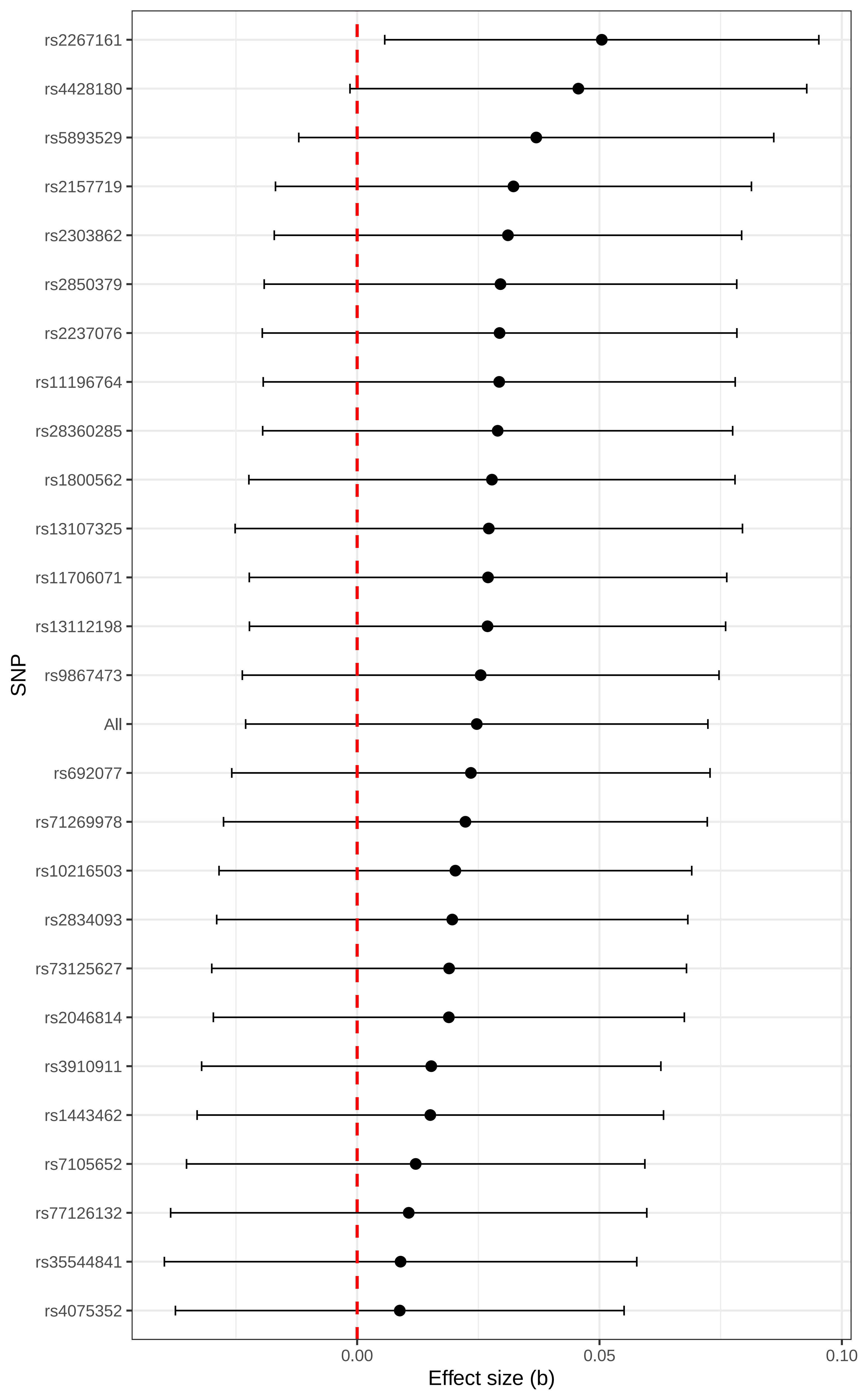

rs2267161
rs4428180
rs5893529
rs2157719
rs2303862
rs2850379
rs2237076
rs11196764
rs28360285
rs1800562
rs13107325
rs11706071
rs13112198
rs9867473
All
rs692077
rs71269978
rs10216503
rs2834093
rs73125627
rs2046814
rs3910911
rs1443462
rs7105652
rs77126132
rs35544841
rs4075352
SNP
0.00
0.05
0.10
Effect size (b)
